# Gene expression profiling in uveal melanoma: technical reliability and correlation of molecular class with pathologic characteristics

**DOI:** 10.1186/s13000-017-0650-3

**Published:** 2017-08-04

**Authors:** Kristen M. Plasseraud, Jeff K. Wilkinson, Kristen M. Oelschlager, Trisha M. Poteet, Robert W. Cook, John F. Stone, Federico A. Monzon

**Affiliations:** 1 Castle Biosciences, Inc., 820 S. Friendswood Drive, Suite 201, Friendswood, TX 77546 USA; 2 Castle Biosciences Laboratory, 3737 N. 7th St, Suite 160, Phoenix, AZ 85014 USA

**Keywords:** Uveal melanoma, Gene expression profiling, Prognosis, Analytic validity

## Abstract

**Background:**

A 15-gene expression profile test has been clinically validated and is widely utilized in newly diagnosed uveal melanoma (UM) patients to assess metastatic potential of the tumor. As most patients are treated with eye-sparing radiotherapy, there is limited tumor tissue available for testing, and technical reliability and success of prognostic testing are critical. This study assessed the analytical performance of the 15-gene expression test for UM and the correlation of molecular class with pathologic characteristics.

**Methods:**

Inter-assay, intra-assay, inter-instrument/operator, and inter-site experiments were conducted, and concordance of the 15-gene expression profile test results and associated discriminant scores for matched tumor samples were evaluated. Technical success was determined from de-identified clinical reports from January 2010 - May 2016. Pathologic characteristics of enucleated tumors were correlated with molecular class results.

**Results:**

Inter-assay concordance on 16 samples run on 3 consecutive days was 100%, and matched discriminant scores were strongly correlated (R^2^ = 0.9944). Inter-assay concordance of 46 samples assayed within a one year period was 100%, with an R^2^ value of 0.9747 for the discriminant scores. Intra-assay concordance of 12 samples run concurrently in duplicates was 100%; discriminant score correlation yielded an R^2^ of 0.9934. Concordance between two sites assessing the same tumors was 100% with an R^2^ of 0.9818 between discriminant scores. Inter-operator/instrument concordance was 96% for Class 1/2 calls and 90% for Class 1A/1B calls, and the discriminant scores had a correlation R^2^ of 0.9636. Technical success was 96.3% on 5516 samples tested since 2010. Increased largest basal diameter and thickness were significantly associated with Class 1B and Class 2 vs. Class 1A signatures.

**Conclusions:**

These results show that the 15-gene expression profile test for UM has robust, reproducible performance characteristics. The technical success rate during clinical testing remains as high as first reported during validation. As molecular testing becomes more prevalent for supporting precision medicine efforts, high technical success and reliability are key characteristics when testing such limited and precious samples. The performance of the 15-gene expression profile test in this study should provide confidence to physicians who use the test’s molecular classification to inform patient management decisions.

**Electronic supplementary material:**

The online version of this article (doi:10.1186/s13000-017-0650-3) contains supplementary material, which is available to authorized users.

## Background

Uveal melanoma (UM) is a rare, intraocular cancer that affects approximately 1600 patients per year in the United States [1]. Most patients are treated with eye-sparing radiotherapy, primarily through plaque brachytherapy or proton beam radiation, while only a small proportion (~10%) will undergo enucleation. Despite the high rate of primary tumor control, ~50% of patients will develop metastatic disease, primarily to the liver, after which prognosis is poor [2]. While the clinicopathologic features assessed in AJCC staging, including tumor size, ciliary body involvement, and extraocular extension, are important factors when assessing metastatic risk, even Stage I-II patients have a 12-30% UM-related mortality rate by 10 years [3, 4]. Because of this, frequent surveillance was generally recommended to monitor all UM patients for disease spread [5, 6].

Gene expression profiles that are associated with low-risk (Class 1A), intermediate-risk (Class 1B), or high-risk (Class 2) outcomes have been shown to provide information useful for risk-tailored surveillance plans [7–9]. Because treatment of the primary tumor is highly effective with plaque radiotherapy, enucleation is not common, and the amount of tumor tissue available for molecular prognostic testing is limited. During the development of the gene expression profiling test for UM prognostication (also known as DecisionDx®-UM), focus was placed on providing patients with a test that was robust and could be run successfully and reproducibly on a very small amount of tissue obtained through a fine needle aspirate biopsy (FNAB), as well as formalin-fixed paraffin embedded (FFPE) tissue from enucleations [8–10].

Recommendations for the development of clinically useful molecular biomarkers have suggested that a biomarker test needs to demonstrate clinical validity, utility, and analytic validity, and that supporting data for each must be transparent and readily available for both physicians and patients [11]. Several widely-used genomic tests for different types of cancer have achieved high levels of evidence for each of these criteria [12–17]. The clinical validity and clinical utility of the 15-gene expression profile test for UM was reported by the Cooperative Ocular Oncology Group (COOG) [9], and the prognostic accuracy of the test has been confirmed in multiple single- and multi-center studies [18–23]. The test’s impact on clinical decision-making for UM patients has also been demonstrated [23, 24]. The focus of this study was to evaluate performance metrics of the test in a CLIA-certified laboratory setting, describe the rate of technical success on both FNAB and FFPE tissue from enucleations, and report correlations with pathological variables.

## Methods

### Tissue acquisition and processing

**Fig. 1:**
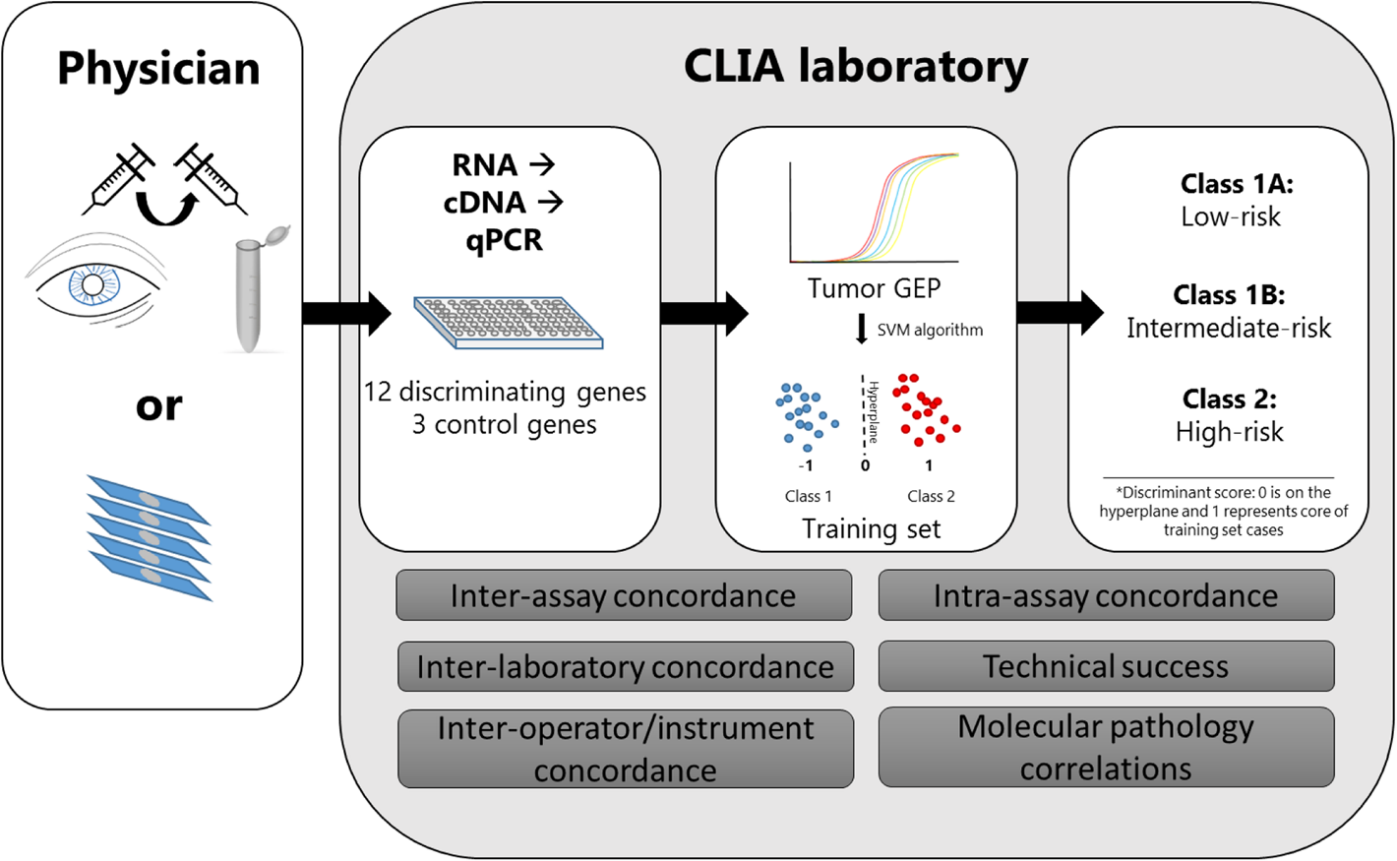
Overview of DecisionDx-UM testing and analytic validation. Fine-needle aspirate biopsies are collected prior to plaque or proton beam clip placement, snap-frozen in RNA stabilization buffer, and shipped frozen. Alternatively, six 5-μm section slides containing tumor tissue from enucleations can be used. RNA is extracted and reverse transcribed into cDNA. The cDNA is pre-amplified followed by qPCR for 12 discriminating genes and 3 control genes. A support vector machine (SVM) algorithm assigns a Class 1 or 2 call comparing the patient sample to a locked-down training set. The summation of *CDH1* and *RAB31* is used to determine Class 1A or 1B subclassification

All samples were acquired during routine clinical testing for risk prognostication in UM patients. For FNAB samples, UM tumor aspirates were frozen in RNase-free RNA stabilization buffer by the treating physician immediately after biopsy and shipped to the Castle Biosciences’ laboratory on dry ice (Fig. 1). All samples were immediately processed with the PicoPure RNA Isolation Kit (Molecular Devices, Sunnyvale, CA). For tumors removed through enucleation, five-micron sections from FFPE tissue were used; the first of six sequential sections were stained with hematoxylin and eosin. Tumor tissue containing at least 80% tumor nuclei density was marked and manually dissected from unstained slides using a sterile scalpel and processed for RNA isolation according to the Ambion RecoverAll Total Nucleic Acid Isolation Kit (Life Technologies Corporation, Grand Island, NY). Reverse transcription of RNA into cDNA was performed using the Applied Biosystems High Capacity cDNA Reverse Transcription Kit (Life Technologies Corporation).

### DecisionDx-UM gene expression profiling and molecular classification

Gene expression profiling was performed as previously described [9, 25]. From 2010 through 2014, quantitative PCR was performed on a 7900HT Fast Real-Time PCR System (Thermo Fisher Scientific), after which it was performed on the current system, a QuantStudio 12K Flex Real-Time PCR System (Applied Biosystems). Complementary cDNA was pre-amplified with 14 cycles and then diluted 20-fold with Tris-EDTA (TE). Fifty microliters of diluted, pre-amplified cDNA was mixed with 50 uL of 2X Taqman Gene Expression Master Mix and loaded in triplicate onto customized microfluidics PCR cards containing the gene-specific primers and probes for the 12 discriminating genes and endogenous control genes (Additional file 1: Table S1).

After PCR amplification, the average Ct values for each triplicate are calculated. The average Ct value for each of the 12 discriminating genes is subtracted from the geometric mean of the control genes to determine the ∆Ct value for each gene. A support vector machine (SVM)-learning algorithm, trained on a training set of UM cases with known gene expression profiles and long-term outcomes, which has been locked since inception, is used to determine classification of test cases. The SVM algorithm places the UM samples within the training set into a hyperplane with *n*-dimensional space and maximizes the hyperplane between the Class 1 and Class 2 specimens. A discriminant score for each sample is generated, which reflects the inverse distance of the patient sample to the hyperplane separating Class 1 and 2 training set samples. The output of the algorithm is either a negative discriminant score (Class 1) or positive discriminant score (Class 2). The discriminant score can theoretically range from >0 to positive or negative infinity, however, only the absolute (positive) value of the discriminant score is reported clinically alongside the respective Class result. A discriminant score is considered reduced confidence if it is <0.1. For Class 1 cases, summation of the *RAB31* and *CDH1* ∆Ct values determined whether the sample was Class 1A or Class 1B based on a mathematical threshold. Class 1B UMs have differential *RAB31*/*CDH1* expression relative to Class 1A tumors.

### Technical reliability studies

De-identified DecisionDx-UM test results from January 2010 through May 2016 were analyzed according to tissue type, successful molecular classification, and resultant class assignment. Institutional review board submission of the technical reliability study was not required as it constitutes technical data on file only, and contains no patient-specific protected health information. As such, this analysis is exempt from the regulatory review requirements as set forth in section 46.101 (b) of 45 CFR 46 [26]. Analytic validation experiments were performed on cDNA generated from the RNA extracted from UM tumor specimens. For these experiments, de-identified residual samples were used. Given that the majority of specimens are obtained via FNAB, most tumor samples are exhausted by performing the 15-gene expression profile test and residual samples are limited. As such, samples used in reproducibility experiments were not consecutive samples, but in each experiment, leftover samples from approximately the same time period were used. Samples were between 1 and 10 weeks old (from date of tissue receipt) and remained frozen after initial processing until reliability experiments.

## Results

### Inter-assay and intra-assay repeatability

**Fig. 2:**
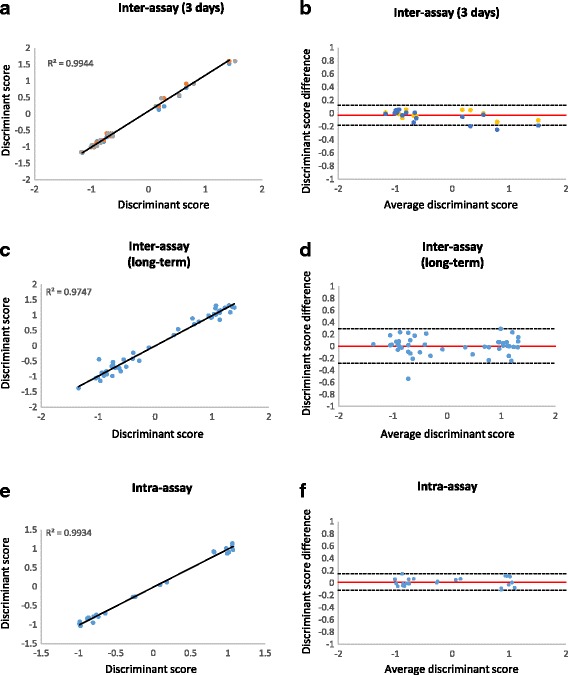
Inter- and intra-assay reliability of DecisionDx-UM. **a** Inter-assay correlation of discriminant scores for 16 samples run on 3 consecutive days. **b** Bland-Altman plot showing the difference in discriminant scores between runs and the 95% confidence interval (*dashed lines*). The estimated bias (or mean difference in discriminant values) is represented as the *red line*. **c** Inter-assay correlation of discriminant scores of 46 samples run twice during a one-year period for proficiency testing or instrument verification. **d** Bland-Altman plot of proficiency testing and instrument verification samples. **e** Intra-assay correlation of discriminant scores of 12 samples tested in duplicate on two cards. **f** Bland-Altman plot showing differences in paired discriminant scores within each card

To evaluate the repeatability of gene expression profiling and class assignment agreement on the same sample between separate PCR runs, DecisionDx-UM testing was performed on 16 samples on three consecutive days, resulting in 48 molecular classifications (Table 1). The class assignment concordance for each sample across all three days was 100% (eight Class 1A, three Class 1B, and five Class 2 tumors). Multiple regression analysis of the discriminant scores from the three runs showed an average R^2^ value of 0.9944 (Fig. 2a). The majority of variation in discriminant scores between runs fell within the 95% confidence interval (−0.1809 to 0.1219), with an estimated bias of −0.0295 (Fig. 2b).

**Table 1:** Summary of results from repeatability and proficiency studies

Study	Design summary	Molecular class calls (n)	Concordance	Bias discriminant scores between replicates (95% CI)	Discriminant score R^2^
Inter-assay (consecutive days)	16 samples, 1 instrument, 2 operators, 3 runs, 3 consecutive days, 1 manufacturing reagent lot	48	100%	−0.0295 (−0.1809-0.1219)	0.9944
Inter-assay (long term)	46 samples run on 2 separate days, 2 instruments, multiple runs, multiple operators, multiple manufacturing reagent lots	92	100%	0.0049 (−0.2816-0.2914)	0.9747
Intra-assay	12 tumors with 2 replicates on 2 plates each, 1 instrument, 1 operator, 3 runs, 3 consecutive days, 1 manufacturing reagent lot	48	100%	0.0145 (−0.1182-0.1472)	0.9934
Inter-site	29 samples, two instruments, two operators, 2 manufacturing reagent lots	58	100%	0.0233 (−0.1939-0.2405)	0.9818
Inter-operator/ instrument	28 samples, 2 instruments, 2 operators, 2 runs, 1 day, 1 manufacturing reagent lot	56	96% (C1 vs C2) 90% (C1A vs C1B)	−0.0540 (−0.3266 -0.2186)	0.9636

In a second reproducibility study, matched duplicate runs of samples used for proficiency testing or instrument verification at six time points during a one-year period were analyzed for inter-assay concordance (Table 1). Overall, reproducibility was evaluated on 46 samples run in duplicate during this one-year interval, and the discriminant scores and molecular classifications were compared for the matched samples. The molecular classifications were 100% concordant for all samples (7 Class 1A, 15 Class 1B, and 24 Class 2 tumors). Regression analysis showed an R^2^ of 0.9747 (Fig. 2c). The estimated bias in discriminant scores was 0.0049 and most variation in discriminant scores fell within the 95% confidence interval (−0.2816 to 0.2914) (Fig. 2d).

Intra-assay repeatability was tested by running two replicates of four samples on the same PCR card, and repeating this experiment on a second card. This was performed with three sets of four samples, resulting in 24 paired molecular classifications from 12 tumor specimens. Class assignment agreement was 100% for the four samples within each card and between the two cards (Table 1; five Class 1A, two Class 1B and five Class 2 tumors). The R^2^ value from correlation analysis was 0.9934 (Fig. 2e). As shown on a Bland-Altman plot (Fig. 2f), all (24/24) of the paired intra-assay discriminant scores had differences that fell within the 95% confidence interval (−0.1182 to 0.1472) and the estimated bias was 0.0145.

**Fig. 3:**
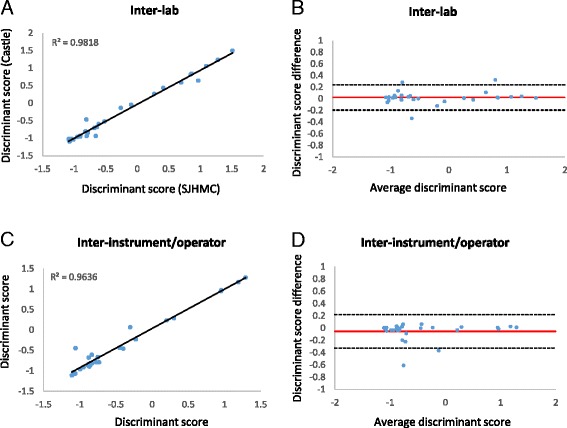
Inter-lab and inter-instrument/operator reliability of DecisionDx-UM. **a** Correlation of 29 paired discriminant scores from samples run at two CLIA-certified laboratories. **b** Bland-Altman plot of the estimated bias (average difference between discriminant scores, *red line*) between labs and the 95% confidence interval (*dashed lines*). **c** Correlation analysis of 28 paired inter-instrument/operator discriminant values. **d** Bland-Altman plot paired inter-instrument/operator discriminant values

Transfer of the test to a new laboratory location afforded the opportunity to test the inter-assay reliability of the test when performed in different laboratories. Twenty-nine samples were run at our CLIA-certified contract laboratory (St. Joseph’s Hospital and Medical Center, Phoenix AZ) and a new Castle Biosciences, Inc. facility (Table 1). The DecisionDx-UM Class calls were 100% concordant between the two laboratories. The R^2^ value of the correlation between discriminant scores was 0.9818 (*p* < 0.001) (Fig. 3a). The estimated bias was 0.0233 and 26/29 (90%) of the discriminant scores had an inter-lab difference that fell within the 95% confidence interval (−0.1939 to 0.2405; Fig. 3b).

Twenty-eight samples were run on two instruments by separate operators to analyze inter-instrument and –operator reliability (Table 1). Two Class 1 calls were discordant for their 1A versus 1B subclassification. One discordant sample had a Class 1B result because the *RAB31*/*CDH1* expression fell just under the subclassification threshold, and its variability fell within the 95% confidence interval for *RAB31*/*CDH1* in reliability experiments. The remaining discordant case had a difference in *RAB31*/*CDH1* between runs that fell outside of this confidence interval and was thus an outlier. Class 1 vs. Class 2 calls were 96% concordant (27/28). The case that was discordant for Class 1 vs. Class 2 did not have any unique characteristic that could potentially explain the discrepancy and thus seems to represent an outlier. As shown in Fig. 3c, correlation analysis of the discriminant scores between the two instruments generated an R^2^ value of 0.9636 (*p* < 0.001). The estimated bias was −0.0540 (95% CI = −0.3266 to 0.2186) and only 1 sample fell outside of this confidence interval (Fig. 3d).

### Technical success of clinical testing

**Fig. 4:**
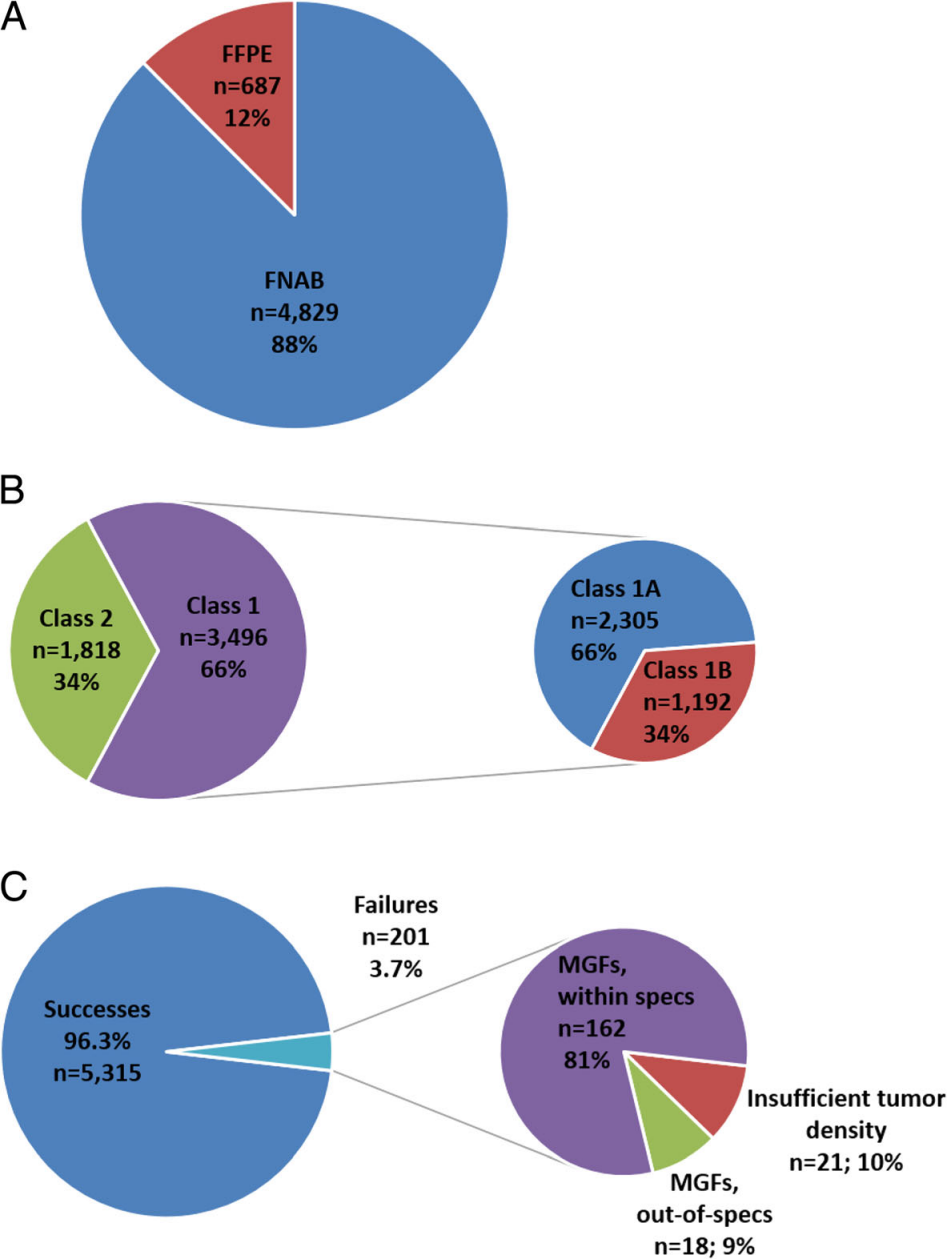
Clinical experience with DecisionDx-UM testing. **a** Proportions of tissue type received for testing. **b** Proportions of Class 1A, 1B, and 2 results in clinical setting from January 2010-May 2016. **c** Technical success of DecisionDx-UM testing and proportions of technical failures due to multi-gene failures, out-of-specification samples, and insufficient tumor density

From January 2010 to May 2016, 5516 tumor specimens were tested. Of these, 4829 (88%) were FNABs and 687 (12%) were FFPE, the vast majority of which were 5-μm sections from enucleations and the remaining as FFPE cell blocks (Fig. 4a, Table 2). Gene expression profiling was successful for 96.35% (5315) of these tumors: 2305 (43.4%) were Class 1A, 1192 (22.4%) were Class 1B, and 1818 (34.2%) were Class 2 (Table 2, Fig. 4b-c).

**Table 2:** Clinical experience of DecisionDx-UM testing

Sample Type	Received	Successfully reported	Multi-gene Failures (MGF)		Insufficient tissue
			within specs	Outside specs	
FNAB (n, % of row)	4829	4678 (97%)	133 (2.7%)	18 (0.3%)	N/A
FFPE (n,% of row)	687	637 (93%)	29 (4%)	N/A	21 (3%)
Total (n, % of row)	5516	5315 (96.3%)	180 (3.3%)		21 (0.4%)

Failure to generate a successful gene expression profile report can be due to i) the failure of multiple control and/or discriminating genes to amplify (multi-gene failure [MGF]), or ii) insufficient tumor cell density (<80%) within a manually dissectable area of FFPE tissue as determined by a pathologist, which results in rejection of the tumor sample prior to testing. Overall, 180 (3.3%) of the samples tested resulted in MGFs and 21 (0.4%) samples had insufficient tumor density (Table 2, Fig. 4c). Eighteen of the MGFs (10%) were related to out-of-specification FNAB specimens, meaning the FNABs were received thawed and/or were not within the acceptable volume range (10-220 uL). Success rates for FNAB and FFPE tissues were similar. Testing was successful for 4678 (97%) out of 4829 FNABs, with only 151 (3%) experiencing MGFs (Table 2). Of the 666 FFPE tumors that passed the quality control assessment and went on to gene expression profile testing, 637 (96%) were successful and 29 (4%) had MGFs.

To determine if long distance shipment has an effect on the reliability of gene expression profiling we evaluated performance on international samples. Since 2010, 218 tumor samples from outside of the United States have been tested; 202 (93%) were fresh-frozen FNABs from Canada and four other countries outside of North America and the remaining 16 (8%) samples were FFPE tissues that came from Canada and two countries outside of North America (data not shown). Only two MGFs occurred (both FNAB samples from Canada), resulting in 99% technical success for internationally shipped specimens.

### Discriminant scores and confidence intervals

**Fig. 5:**
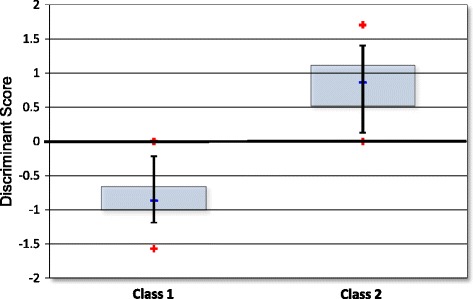
Distribution of discriminant scores. The distribution of discriminant scores that have been reported clinically. The output of the SVM algorithm is negative for Class 1, but reported as a positive number. The 50th percentile is represented by the *blue box* and the *black bars* indicate the 90th percentile. The *blue line* is the median of each group and the red stars indicate the minimum and maximum observed scores of each Class

The discriminant score associated with the DecisionDx-UM Class reflects the inverse distance of the patient sample to the hyperplane. As of May 2016, the discriminant scores have ranged from −1.56808 to +1.70482 (Fig. 5). The average discriminant score for Class 1 was −0.81033 (95% CI = −0.256 to −1.365). The average discriminant score for Class 2 was 0.816144 (95% CI = 0.0528 to 1.567). A score between −0.1 to 0.1 is considered of reduced confidence and is reported clinically as such. However, a discriminant score with reduced confidence has not been associated with a different outcome than a normal confidence score [25], and the vast majority (~97%) of the discriminant scores reported clinically have been reported within the normal confidence range.

### Pathologic characteristics of DecisionDx-UM-tested tumors

Associated de-identified pathological data was available for 527 FFPE tumors from enucleations tested between January 2010 and May 2016. The pathologic characteristics of these tumors are described in Table 3, with statistical analysis of correlation with molecular class presented in Table 4. Eighty-eight tumors (17%) were Class 1A, 201 (38%) were Class 1B, and 238 (45%) were Class 2. Class 2 tumors had significantly greater largest basal diameters (LBDs) than Class 1 tumors (*p* < 0.0001 by Mann-Whitney test). Compared to Class 1A tumors, Class 1B tumors also had significantly greater LBDs (median of 14 mm vs 11.5 mm, *p* = 0.008 by Mann-Whitney test). Similarly, Class 2 tumors were significantly thicker than Class 1 tumors (median of 9.45 mm vs 7, *p* < 0.0001), and within Class 1, Class 1B tumors were significantly thicker than Class 1A tumors (median of 8 mm vs 6 mm, *p* = 0.001). A Class 1 signature was associated with predominantly spindle cell type, while a Class 2 signature was significantly associated with a mixed or predominantly epithelioid cell type (*p* < 0.0001 by Fisher’s exact). Class 1A and 1B tumors had similar cell morphologies, while Class 2 tumors were significantly associated with a mixed/predominantly epithelioid cell type compared to Class 1B tumors (*p* = 0.0002 by Fisher’s exact). There was no difference in ciliary body involvement between Class 1A and 1B tumors, but Class 2 tumors were more likely to have ciliary body involvement than Class 1A and 1B tumors (*p* < 0.0001 by Fisher’s exact). There was no significant association with any molecular class and extra-ocular extension.

**Table 3:** Morphologic characteristics and correlation with molecular class result in enucleated cases

	Class 1A (*n* = 88, 17%)	Class 1B (*n* = 201; 38%)	Class 2 (*n* = 238, 45%)
Largest basal diameter (mm), median (range)	11.5 (1.5-28)	14 (1.1-35)	16 (1.4-39)
≤12 mm (n, %)	47 (53%)	75 (37%)	57 (24%)
> 12 mm (n, %)	29 (33%)	109 (54%)	165 (69%)
Not addressed (n, %)	12 (14%)	17 (9%)	16 (7%)
Tumor height (mm), median (range)	6 (0.4-20)	8 (0.5-29)	9.45 (1-36)
≤ 5.5 mm (n, %)	34 (39%)	47 (23.5%)	51 (21%)
> 5.5 mm (n, %)	42 (47%)	135 (67%)	166 (70%)
Not addressed	12 (14%)	19 (9.5%)	21 (9%)
Cell type (n, %)			
Spindle predominant	40 (45.5%)	92 (46%)	65 (27%)
Mixed/epithelioid predominant	40 (45.5%)	102 (51%)	158 (66%)
Not addressed	8 (9%)	7 (3%)	15 (6%)
Ciliary body involvement (n,%)			
No	35 (40%)	104 (52%)	84 (35%)
Yes	23 (26%)	45 (22%)	111 (47%)
Not addressed	30 (34%)	52 (26%)	43 (18%)
Extra-scleral/ocular extension (n,%)			
No	59 (67%)	145 (72%)	176 (74%)
Yes	14 (16%)	34 (17%)	42 (18%)
Not addressed	15 (17%)	22 (11%)	20 (8%)

**Table 4:** Statistical comparisons between molecular class and tumor pathology in enucleated cases

	Class 1 vs. Class 2	Class 1A vs. Class 1B	Class 1B vs. Class 2	Class 1A vs. Class 2
Largest basal diameter^a^	*p* < 0.0001	*p* = 0.008	*p* = 0.0001	*p* < 0.0001
Tumor height^a^	*p* < 0.0001	*p* = 0.0015	*p* = 0.0085	*p* < 0.0001
Cell type^b^	*p* < 0.0001	n.s.	*p* = 0.0002	*p* = 0.001
Ciliary body involvement^b^	*p* < 0.0001	n.s.	*p* < 0.0001	*p* < 0.0001
Extra-scleral/ocular extension	n.s.	n.s.	n.s.	n.s.

^a^Mann-Whitney test; ^b^Fisher’s exact test

## Discussion

Given the advances in molecular cancer diagnostics in the last decade, recommendations have been established to guide careful, thorough assessments of these tests [11, 27]. One of the major criteria for a clinically useful, well-validated assay is that the test must demonstrate analytic validity, which includes reliability and reproducibility of the test to measure the intended molecular analytes [11, 27]. In this study, we report on the analytic validity of the 15-gene expression profile test (DecisionDx-UM) as performed in a CLIA-certified clinical laboratory. Reproducibility and reliability were demonstrated through i) inter-assay concordance, ii) inter-assay concordance of samples used for proficiency testing and instrument verification throughout one year, iii) intra-assay concordance, iv) inter-lab concordance between two CLIA-certified laboratories, and v) inter-instrument/operator concordance. Molecular classifications by DecisionDx-UM were 100% concordant for four of these assessments, with only 3 discordant specimens out of the total 143 samples in the analytic experiments. The reported discriminant scores for the same tumor sample were highly correlated, as reflected by R^2^ values above 0.96 for the inter-assay, intra-assay, inter-instrument, and inter-lab experiments. Furthermore, the run-to-run, lab-to-lab, instrument-instrument, and intra-run variabilities between discriminant scores were all within acceptable limits that would not impact patient care. These results demonstrate that the DecisionDx-UM class assignment for the same tumor specimen is consistent within the same PCR card and when run on separate days in different PCR cards, even with several weeks in between runs. To our knowledge, this is the only report of analytic validity for any prognostic test for UM. Comparable analytic validation has not yet been reported for fluorescence in situ hybridization (FISH), microsatellite analysis (MSA), single nucleotide polymorphism (SNP) arrays, or multiplex-ligation probe amplification (MLPA), all of which can be used to detect monosomy 3 and other chromosomal copy number changes that have been associated with UM metastasis, or next-generation sequencing to detect mutations and chromosomal aberrations to estimate UM prognosis.

Gene expression profiling is one of the clinically significant variables for disease prognostication recommended by the 2017 AJCC staging guidelines [28], and as such, the DecisionDx-UM test is routinely used across the United States and has been clinically available since 2010. The prognostic test was developed after two distinct molecular subtypes of UM were identified and shown to have correlation with outcomes [7, 10, 29, 30]. A 15-gene RT-PCR test was developed to identify these UM molecular subtypes and prospectively validated in multiple studies [8, 9, 20, 21, 23]. As eye-sparing treatments are frequently utilized in the contemporary management of UM and enucleations are less common, the standard practice for physicians utilizing DecisionDx-UM is to collect a biopsy prior to or at the time of radioactive plaque or proton beam clip placement. Due to the lack of residual tissue aside from that obtained during biopsy, successful gene expression profiling on a single biopsy is critical. While the technical success of the 15-gene expression profile assay has been previously reported as ranging from 95 to 99% [8, 9, 19], many of the samples in those studies were tested in the research laboratory that developed the assay, which was then licensed by Castle Biosciences, Inc. in 2009. In this report, we demonstrate that 96.4% of 5516 samples that have been clinically tested at Castle Biosciences’ laboratory generated successful molecular classification reports, establishing the consistency of technical success from the test. Of the unsuccessful tests, 39 out of 201 (19%) were due to samples outside of quality control specifications (for FNABs, incorrect volume and/or not frozen, *n* = 18; for FFPE tissues, insufficient tumor volume, *n* = 21). Thawed samples and those with insufficient/excessive volume have been previously reported to be associated with technical failures of gene expression profiling [8]. The technical success rate of DecisionDx-UM testing is substantially higher than what has been previously reported for other molecular methods used in UM prognostication, including FISH, MSA, and array CGH, which range from 50 to 87% in much smaller sample sets [31–34].

Of the 5315 successful tests reported, 43.4% were Class 1A, 22.4% were Class 1B, and 34.2% were Class 2. Overall, these proportions are similar to class those reported in the Cooperative Ocular Oncology Group (COOG) study, which were 47% Class 1A, 13%, Class 1B and 40% Class 2 [9]. Likewise, other single- and multi-center studies that analyzed subsets of the clinically tested patients reported here have shown similar proportions of Class 1A, 1B, and 2 results [21, 23, 35].

Of the enucleation specimens tested for which there were associated pathology data, the proportion of Class 1 patients shifted to be predominately Class 1B vs. Class 1A, and the proportion of Class 2 tumors was also increased compared to the total clinical population. Given the significantly increased LBDs and thicknesses of enucleation specimens (i.e. those that necessitated removal of the globe), an increase in Class 2 and Class 1B tumor classification is not unexpected. Importantly, there were no significant differences between Class 1A and 1B tumors in terms of cell morphology, ciliary body involvement, or extraocular extension, underscoring the utility of molecular testing to delineate risk in these tumors that otherwise share similar pathologic features. Overall, the pathology and molecular class data in our clinically tested cohort reflect published reports that greater LBD and tumor thickness tend to be clinicopathologic features associated with more aggressive tumors [9, 20, 22, 23], and these riskier phenotypes are most frequently seen in Class 1B or Class 2 tumors. An advantage of gene expression profiling is that it reflects objective, intrinsic tumor biology, whereas measurements of LBD in particular can be subjective due to variation between observers and in techniques used for size measurement [36]. Additionally, cytopathologic analysis can be impaired by a high rate of insufficient cellularity from FNABs [19]. Several studies have shown that GEP is the most significant independent prognostic factor for metastatic risk when compared to clinicopathologic features, including LBD [9, 18–20, 22, 23, 37].

## Conclusions

In summary, the results of these analytic performance data demonstrate the reproducibility and reliability of the DecisionDx-UM test. This is confirmed by the high correlation of discriminant scores and concordance of molecular classifications on samples subjected to repeat testing. The robust high technical success rate of the test on even small amounts of tissue obtained by FNAB has been maintained from the test’s original development through clinical implementation and testing of more than 5000 patients, and represents an important aspect of testing, given that patients who receive eye-sparing radiotherapy, usually do not have the opportunity to be biopsied again after treatment.

## Additional file

Additional file 1: Table S1. 12 Discriminating genes in DecisionDx-UM. (DOCX 16 kb)

## Abbreviations

COOG: Collaborative Ocular Oncology Group
FFPE: Formalin-fixed, paraffin-embedded
FISH: Fluorescence in situ hybridization
FNAB: Fine needle aspirate biopsy
LBD: Largest basal diameter
MGF: Multi-gene failure
MLPA: Multiplex ligation probe amplification
MSA: Microsatellite analysis
UM: Uveal melanoma
