# Supplementary material for: Gene expression profiling in uveal melanoma: technical reliability and correlation of molecular class with pathologic characteristics
Source: Diagn Pathol. 2017 Aug 4;12:59. doi: 10.1186/s13000-017-0650-3 (PMC5545042; doi:10.1186/s13000-017-0650-3)
Supplement: Additional file 1: Table S1. — 12 Discriminating genes in DecisionDx-UM. (DOCX 16 kb) [file 13000_2017_650_MOESM1_ESM.docx]

| Gene Symbol | Gene Name | Function |
| --- | --- | --- |
| *CDH1* | Cadherin 1 (E-Cadherin) | Cell-cell adhesion; tumor suppressor |
| *ECM1* | Extracellular matrix protein 1 | Glycoprotein that functions in cell proliferation, differentiation, angiogenesis |
| *HTR2B* | 5-hydroxytryptamine receptor 2B | G protein-coupled receptor for serotonin |
| *RAB31* | RAB31, member RAS oncogene family | Small GTPase that regulates intracellular membrane trafficking |
| *EIF1B* | Eukaryotic translation initiation factor 1B | Component of the pre-initiation complex and contributes to mRNA scanning |
| *FXR1* | FMR1 autosomal homolog 1 | RNA-binding protein that may regulate intracellular transport and translation of mRNAs |
| *ID2* | Inhibitor of DNA binding 2, dominant negative helix-loop-helix protein | Transcriptional regulator that negatively regulates cell differentiation |
| *LMCD1* | LIM and cysteine rich domains 1 | Zinc-finger co-transcription factor |
| *LTA4H* | Leukotriene A4 hydrolase | Enzyme that catalyzes leukotriene B4 and processes inflammatory peptides |
| *MTUS1* | Microtubule associated tumor suppressor 1 | Tumor suppressor that inhibits ERK2 activation and cell proliferation |
| *ROBO1* | Roundabout guidance receptor 1 | Transmembrane receptor for SLIT1; plays a role in axon guidance |
| *SATB1* | SATB homeobox 1 | Binds nuclear matrix; recruits chromatin-remodeling factors and organizes chromatin architecture |

**Additional file 1: Table S1**. 12 Discriminating genes in DecisionDx-UM
